# Non-operative versus reverse shoulder arthroplasty for the treatment of 3- or 4-part proximal humeral fractures: A systematic review and meta-analysis

**DOI:** 10.1016/j.jcot.2025.102982

**Published:** 2025-03-22

**Authors:** Victor Yan Zhe Lu, Halia Shah, Zainab Alshaber, Aaron Limonard, Peter Domos

**Affiliations:** a Department of Orthopaedics and Traumatology, Chinese University of Hong Kong, Hong Kong; b St George's, University of London, SW17 0RE, United Kingdom; c University of Glasgow Medical School, Glasgow, G12 8QQ, United Kingdom; d Department of Trauma and Orthopaedics, Barnet and Chase Farm Hospital, Royal Free NHS Foundation Trust, London, NW3 2QG, United Kingdom

**Keywords:** Proximal humerus fracture, Reverse shoulder arthroplasty, Systematic review, Elderly, Non-operative

## Abstract

**Background:**

Proximal humerus fractures (PHFs) are a common injury experienced by elderly patients, however there is no consensus regarding the best treatment option. Recently, the use of reverse shoulder arthroplasty (RSA) in elderly patients with complex fractures is increasing. This systematic review and meta-analysis will compare the outcomes between RSA and non-operative treatment in 3- or 4-part PHFs in the elderly.

**Methods:**

This study was conducted according to the PRISMA statement protocol and registered in PROSPERO (CRD42023439647). Searches on four databases (Medline, Embase, Web of Science, and Cochrane Library) were performed, and comparative studies which compared the outcomes of using RSA with conservative management were included. Demographic data, patient related outcome measures (PROMs), and complications rates were collected. Data were pooled using a random-effects model. Heterogeneity was determined using the I^2^ statistic and Cochran's Q test.

**Results:**

Six studies involving 439 patients (mean age 79.0 years old; 12.1 % male) were analysed. The average Charlson co-morbidity index (CCI) was 3.74 and follow-up time was 26.0 months. Compared to the non-operative cohort, the RSA cohort had better VAS scores (1.0 versus 0.575; p = 0.047), Constant-Murley scores (66.3 versus 71; p = 0.114), active forward flexion (121.5° versus 100°; p = 0.023; I^2^ = 35 %), external rotation (34.8° vs 23.1°; p = 0.020), and internal rotation (Constant score 5.44 versus 4.28; p = 0.169). There is no difference in the overall risk of complications (8.2 % versus 6.0 %; RR = 1.00; p = 0.993), but those treated by RSA have a higher risk of needing subsequent revision surgery (3.7 % versus 2.8 %; p = 0.640; I^2^ = 25 %).

**Conclusion:**

In the short-term, elderly patients with complex PHFs treated with RSA may have decreased pain, increased Constant-Murley scores, and increased ROM compared with patients treated non-operatively, at the expense of a higher risk of needing subsequent surgery. However, moderate between-study heterogeneity in effect sizes and the retrospective nature of included studies may limit the clinical applications of conclusions obtained in this review.

**Level of evidence:**

III.

## 1 Introduction

Proximal humerus fractures (PHFs) are the 2nd most common fractures in the upper limb^1^ and the 3rd most common type of osteoporotic fractures in the elderly.^2^ PHFs usually occurs in either young patients, representing high energy trauma, or in the elderly, representing low energy fragility fractures.^3^ With a lifetime risk of 13 % in those over 50 years old,^3^ the incidence of PHFs is predicted to triple by 2030 due to an aging population.^2^

The PROFHER randomised controlled trial (RCT) concluded that there are no significant differences in patient reported outcome measures (PROMs) and mortality rate between surgical and non-surgical treatment for patients with PHFs up to five years follow-up.^4^^,^^5^ Given the risks of peri- and post-operative complications and the high cost of surgery, this conclusion has far reaching implications for clinical practice. However, there are important limitations which may be addressed in the upcoming PROFHER-2 trial.^6^ Neer four-part fractures only accounted for 4.4 % of the sample size, and the cohort included any patient over the age of 18. Furthermore, patients were excluded from the trial if they had a ‘clear indication for surgery’, biasing the results.

As shown by a recent meta-analysis, differences in fracture type and surgical techniques leads to outcomes that differ from those obtained from generalised analyses of all types of PHFs, patients of all ages, and all surgical treatments.^7^ Rather than coming to a conclusion that surgery is inferior or superior to non-operative treatment, it is important to ascertain if there are specific surgical techniques that will benefit specific subgroup of patients.

In the elderly, patients’ osteoporotic bone lends itself to more complex fracture patterns as well as high failure rates of up to 50 % using osteosynthetic techniques.^8^^,^^9^ The PROFHER trial used open reduction and internal fixation (ORIF) in 83 % of the cohort.^4^ This may contribute to why the PROFHER trial concluded that operative treatment showed no significant benefit over non-operative treatment. Recently, there has been an increasing trend towards the use of reverse shoulder arthroplasty (RSA) in elderly patients with complex fractures, as shown by the 2021 UK National Joint Registry (NJR).^10^ However, some studies report a four times greater odds of a postoperative complication compared to hemiarthroplasty (HA),^11^ and no significant improvement in PROMs compared to non-operative treatment.^6^ There is therefore clinical uncertainty regarding the effectiveness of RSA for 3 or 4-part PHFs in the elderly.

A meta-analysis published in 2024 compared the outcomes between arthroplasty and conservative treatment, however their inclusion criteria included patients treated with all kinds of arthroplasty techniques, including hemiarthroplasty.^12^ There has not yet been a comprehensive review of the outcomes of RSA compared to non-operative management in the elderly. Hence, the purpose of this systematic review is to evaluate the clinical and functional outcomes of RSA versus non-operative treatment for comminuted PHFs in the elderly.

## 2 Methods

a. Study Selection

This review followed guidelines for the Preferred Reporting Items for Systematic Reviews and Meta-Analysis (PRISMA), and was registered in the PROSPERO database (CRD42023439647). Two reviewers searched the following four databases from inception to October 2023: Medline, Embase, Web of Science, and Cochrane Library. The search contained variations of the terms ‘proximal humerus fracture’ and ‘reverse shoulder arthroplasty’. Supplementary Table 1 provides the full search strategy.

Studies found in the search were imported into Mendeley and de-duplicated. After title and abstract screening, two reviewers independently assessed the full text of each manuscript. The senior author was contacted in case of any conflicts. The PICOS model (Population, Intervention, Comparison, Outcome, Study type) was used to define the inclusion and exclusion criteria. Only comparative studies were included, which compared the outcomes of using RSA with non-operative management to treat 3- or 4-part PHFs in populations over the age of 65. Patients with pathological fractures, joint infections, neurological lesions, or severe arthritis were excluded. The detailed criteria is shown in Supplementary Table 2.

b. Data Extraction

Data collection was performed using tables created in a Microsoft Excel spreadsheet. In each study, data was split into the following categories.

1. Study characteristics and demographics: Cohort size, %male, BMI, age, Charlson comorbidity index (CCI), laterality, follow-up time, fracture classification.
2. PROMs
3. Range of motion (ROM), including forward flexion (active and passive), extension, abduction, adduction, external and internal rotation.
4. Radiographical outcomes such as radiographic lucency, tuberosity healing, scapular notching
5. Complications, such as reoperation rate, 1-year mortality, non-union, infection, nerve injury, dislocation.
c. Data Analysis

Clinical outcomes from the following PROMs were eligible to be pooled: American Shoulder and Elbow Surgeons (ASES) score, Constant-Murley, visual analogue scale (VAS), disabilities of arm, shoulder & hand (DASH) score, Patient-Reported Outcomes Measurement Information System (PROMIS) score, 12-Item Short Form Survey, Subjective Shoulder Value (SSV) score. ROM quantitative data and complication rate were pooled where appropriate. Non-union was defined as the lack of union after nine months without signs of healing for three months or more.^13^ The need for subsequent surgery was compared between the RSA and non-operative cohorts. Amongst the reported PROMs, a higher absolute value means a better clinical or functional outcome. Exceptions include negatively worded concepts such as the VAS pain score, the PROMIS pain score, and the PROMIS depression score, whereby a lower absolute value indicates a better score. Internal rotation was quantified using the Constant-Murley scale,^14^ which gives a numeric score based on the level of spine reached during internal rotation (lateral thigh = 0, buttock = 2, lumbosacral junction = 4, waist = 6, 12th dorsal vertebra = 8 and interscapular region = 10 points). The CCI is a predictor of ten-year mortality for a patient with multiple co-morbidities. Developed in 1987, it is widely used in clinical research to stratify patients so that comorbidities which can affect overall survival can be controlled for. The index produces a co-morbidity-age combined risk score, which is then converted to give a predicted 10-year survival percentage.^15^

Meta-analyses were carried out using the program RStudio. A minimum of two studies which reported the same outcome measure were needed to produce a forest plot. A random-effects model with inverse-variance weighting was used since there were predicted heterogeneity between studies. When presenting the difference in means between two independent groups, the standardised mean difference (SMD) was reported. By standardising the value, this allows the difference to be demonstrated in units of standard deviations (SD). The raw difference in means is divided by the pooled SD to obtain the SMD. This allows studies that measure an outcome measure in different ways to be compared. When the standard deviation is not reported, an estimation method published by Wan et al.^16^ which takes into account the sample size was used to estimate it. This estimator can also be adjusted to utilise the interquartile range if provided. Missing data were attempted to be retrieved by contacting the corresponding author of each study.

To assess heterogeneity, the I^2^ statistic^17^ and Cochran's Q test^18^ were used. Low heterogeneity was defined as I^2^ ≤ 25 %, moderate heterogeneity as 25 %<I^2^ ≤ 50 %, significant heterogeneity as I^2^>50 %. Funnel plots and Egger's regression test was performed to look for publication bias. Statistical significance was defined if p < 0.05.

d. Risk of Bias

Risk of bias was assessed using Cochrane's RoB 2.0 tool for randomised trials containing five domains^19^: A three-point Likert scale was used for each category – low concern, some concerns, or high risk of bias. Cochrane's ROBINS-I tool was used for non-randomised trials, containing seven domains^20^: A four-point Likert scale was used for each category – low, moderate, high, or critical bias. Summary tables from risk of bias assessments are presented using the ‘robvis’ package in RStudio.^21^

## 3 Results

**Fig. 1:**
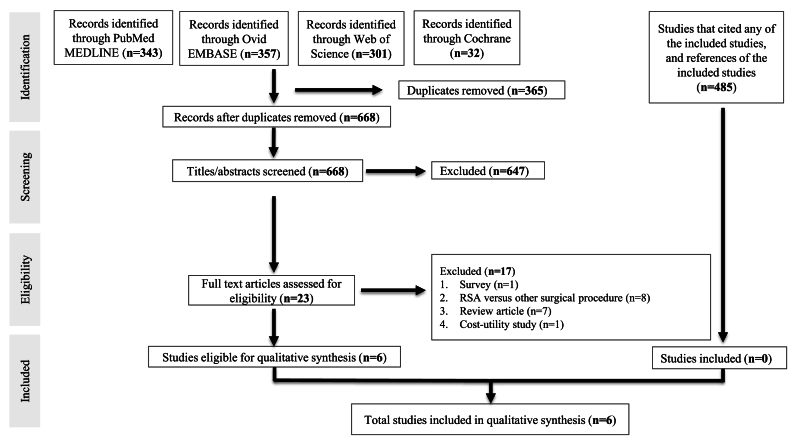
PRISMA Diagram.

The initial search identified 1033 studies from four databases. After the removal of 365 duplicates, 668 studies were included for title and abstract screening. 23 full texts were screened for eligibility, and six were included for data synthesis. The full study selection process is shown in Fig. 1.

a. Demographics

Table 1 shows the study design and patient demographics. All studies apart from one^1^ were retrospective cohort studies. All studies were published within the last six years. The total patient population is 439, with 189 being treated with RSA and 250 being treated non-operatively. The mean age at the time of treatment was 79.04 years old and an average of 12.1 % were male. All studies except one^22^ reported the CCI with an average value of 3.74. Only two studies reported time from injury to treatment.^1^^,^^23^ Chivot et al.^22^ was the only study to report BMI (26.51) and laterality (58.2 % right shoulder). Chivot et al. and Lopiz et al. specified whether the injury was in the dominant limb; 57.7 % of patients had PHFs in their dominant limb. All studies included Neer 3- or 4-part PHFs apart from Samborski et al. who included patents with AO/OTA type 11C (4-part) fractures. The average follow-up time was 26.0 months.

b. Interventions

**Table 1:** Demographics.

Author	Total Patient No.	Cohorts	Age (at surgery)	Sex (% male)	BMI	Follow-up (months)
Chivot et al., 2019	60	RSA: 28	77 (70–92)	21.4	27.19	31.8 (24–52)
		Non-Op: 32	79.2 (70–92)	6.3	25.83	32.1 (24–43)
Lopiz et al., 2019	59	RSA: 29	82 ± 3.4	13	N/A	12
		Non-Op: 30	85 ± 4.8	14		
Rotman et al., 2018	145	RSA: 62	82.2	22.6	N/A	12
		Non-Op: 83	83.7	19.3		
Roberson et al., 2017	39	RSA: 20	71	5	N/A	53
		Non-Op: 19	71	21		29
Samborski et al., 2022	65	RSA: 24	77	4.2	N/A	12
		Non-Op: 41	77	9.8		
Haws et al., 2023	71	RSA: 26	76.8	0	N/A	12
		Non-Op: 45	76.4	8.9		

RSA: reverse shoulder arthroplasty; BMI: body mass index.

Detailed descriptions of surgical and non-operative management were provided in three studies.^1^^,^^22^^,^^23^ Surgical technique consisted of the usage of general anaesthesia in all patients, in the beach-chair position, with a deltopectoral approach. The glenoid baseplate was placed inferiorly on the glenoid to minimise scapular notching. Cancellous bone graft from the resected humeral head was used by Lopiz et al.^1^ No study reported the total operation time or blood loss. Rehabilitation in both the non-surgical and surgical group were the same. This consisted of immobilisation in a sling for 3–6 weeks, followed by passive then active mobilisation.

c. PROMs

**Fig. 2:**
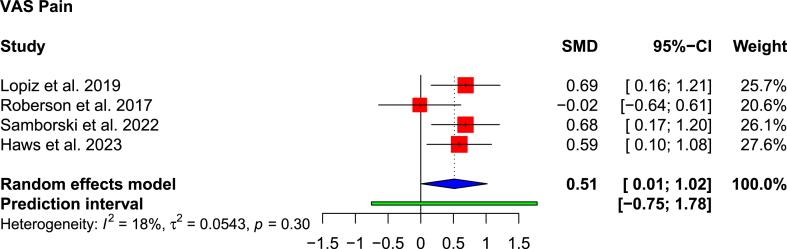
Forest plot showing pooled VAS Pain scores.

**Fig. 3:**
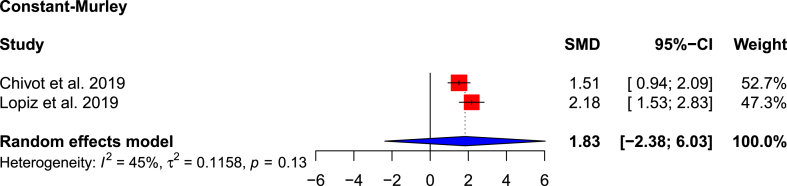
Forest plot showing pooled Constant-Murley scores.

All studies apart from Rotman et al.^24^ presented PROMs (Table 2). VAS pain scores were provided by four studies,^1^^,^^13^^,^^23^^,^^25^ with significantly better pain scores in the RSA cohort (1.0 versus 0.575; SMD: 0.514; 95 % CI: 0.012 to 1.016; p = 0.047; I^2^ = 18 %) (Fig. 2). Roberson et al. reported a better pain score in the non-operative cohort (1.1 vs 1.5). In the RSA cohort, those who had early RSA (<30 days from injury) had more severe pain (VAS score 2) than those who had delayed RSA (>30 days from injury) (VAS score 1.3). Constant-Murley scores were superior in the RSA cohort than the non-operative cohort (66.3 versus 71.9; SMD: 1.827; 95 % CI: −2.376-6.031; p = 0.114; I^2^ = 45 %) (Fig. 3). A significantly better PROMIS physical function score was reported in the RSA cohort by Samborski et al.^25^ (46 versus 37.9; p = 0.01) and Haws et al.^13^ (44.9 versus 37.6; p = 0.02). Haws et al.^13^ also reported a significantly better PROMIS pain score in the RSA cohort (51.7 versus 58.1; p = 0.02). DASH was reported by two studies^1^^,^^22^; only one study reported SF-12,^1^ EQ-5D,^1^ SSV,^22^ which is not adequate to produce a forest plot.

d. Range of Motion

**Table 2:** Clinical outcome scores.

Author		ASES	Constant-Murley	SF-12	EQ-5D	DASH	VAS	SSV	PROMIS pain	PROMIS physical	PROMIS depression
Chivot et al., 2019	*RSA*	N/A	82.1	N/A	N/A	38.7	No Pain: 15 (53 %)	73	N/A	N/A	N/A
	*Non-Op*		76.8			31.2	No Pain: 19 (59 %)	67			
	*Difference*		5.3			7.5	6 %	6			
	*p-value*		0.03			0.11	0.47	0.47			
Lopiz et al., 2019	*RSA*	N/A	61.7 ± 12.1	37.1	0.92 ± 0.13	20.7 ± 13.9	0.9 ± 0.9	N/A	N/A	N/A	N/A
	*Non-Op*		55.7 ± 12.4	36.4	0.89 ± 0.14	28.8 ± 19.6	1.6 ± 2.2				
	*Difference*		6.0	0.7	0.03	8.1	0.7				
	*p-value*		0.071	0.709	0.319	0.075	0.011				
Rotman et al., 2018	*RSA*	N/A	N/A	N/A	N/A	N/A	N/A	N/A	N/A	N/A	N/A
	*Non-Op*										
	*Difference*										
	*p-value*										
Roberson et al., 2017	*RSA*	72	N/A	N/A	N/A	N/A	1.5	N/A	N/A	N/A	N/A
	*Non-Op*	72					1.1				
	*Difference*	0					0.4				
	*p-value*	0.70					0.51				
Samborski et al., 2022	*RSA*	N/A	N/A	N/A	N/A	N/A	0.1	N/A	52.1	46	47.1
	*Non-Op*						0.6		58.4	37.9	49.7
	*Difference*						0.5		6.3	8.1	0.6
	*p-value*						<0.01		0.07	0.01	0.24
Haws et al., 2023	*RSA*	N/A	N/A	N/A	N/A	N/A	0.1	N/A	51.7	44.9	47.7
	*Non-Op*						0.7		58.1	37.6	52.1
	*Difference*						0.6		6.4	7.3	4.4
	*p-value*						0.02		0.02	0.02	0.16

ASES: American Shoulder and Elbow Surgeons; SF-12: 12-Item Short Form Survey; DASH: Disabilities of the Arm, Shoulder and Hand; SSV: Subjective Shoulder Value; VAS: Visual Analogue Scale; EQ-5D: EuroQol 5 Dimension; PROMIS: Patient Reported Outcomes Measurement Information System.

**Fig. 4:**
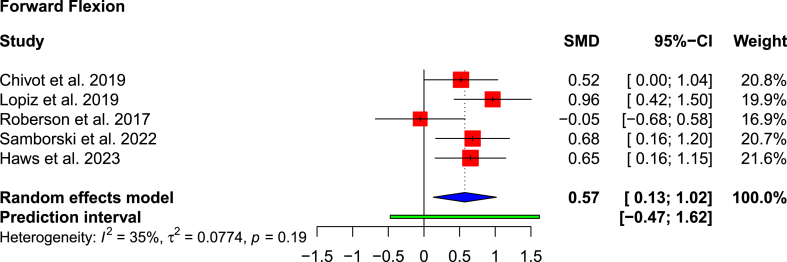
Forest plot showing pooled forward flexion movement.

**Fig. 5:**
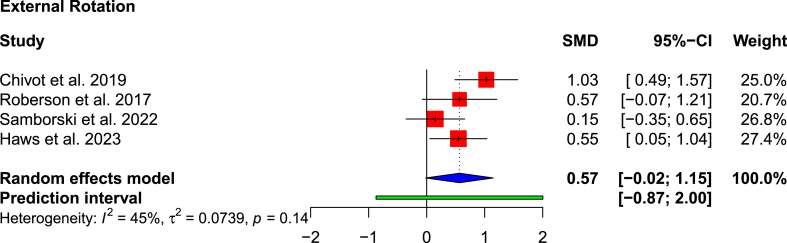
Forest plot showing pooled external rotation movement.

All studies apart from Rotman et al.^24^ reported ROM outcomes (Table 3). The remaining five studies reported significantly greater active forward flexion in the RSA cohort (121.5°) than in the non-operative cohort (100.0°), with a SMD of 0.57 (95 % CI: 0.13–1.02; p = 0.023; I^2^ = 35 %) (Fig. 4). Two studies presented passive forward flexion measurements after treatment^13^^,^^25^; in both, the RSA cohort had greater passive forward flexion (p < 0.01). External rotation was reported by five studies, but only the results from four could be pooled.^13^^,^^22^^,^^23^^,^^25^ The magnitude was greater in the RSA cohort (34.8° vs 23.1°), but was only statistically significant in two studies^13^^,^^22^ (SMD: 0.57; 95 % CI: −0.02 to 1.15; p = 0.020; I^2^ = 45 %) (Fig. 5). Lopiz et al.^1^ reported external rotation based on descriptive terminology, e.g. whether the hand could be placed behind or in front of the head.

**Table 3:** Range of motion.

Author		Forward flexion (active)	Forward flexion (passive)	Abduction	External Rotation	Internal rotation (CM score)^a^
Chivot et al., 2019	*RSA*	110 (90–130)	N/A	N/A	19 (0–40)	5.36
	*Non-Op*	98 (70–120)			9 (0–40)	3.88
	*Difference*	12			10	N/A
	*p-value*	<0.01			<0.01	N/A
Lopiz et al., 2019	*RSA*	114.3	N/A	111.2	Complete ER: 4 (14 %)	5.52
	*Non-Op*	95.5		94.5	Complete ER: 1 (3.3 %)	4.67
	*Difference*	18.8		16.7	N/A	N/A
	*p-value*	N/A		N/A	N/A	N/A
Rotman et al., 2018	*RSA*	N/A	N/A	N/A	N/A	N/A
	*Non-Op*					
	*Difference*					
	*p-value*					
Roberson et al., 2017	*RSA*	119	N/A	N/A	31	N/A
	*Non-Op*	120			23	
	*Difference*	1			8	
	*p-value*	0.87			0.06	
Samborski et al., 2022	*RSA*	133	147.9	N/A	44.1	N/A
	*Non-Op*	93	116.0		37.9	
	*Difference*	40	31.9		6.2	
	*p-value*	<0.01	<0.01		0.56	
Haws et al., 2023	*RSA*	131.4	145.4	N/A	45	N/A
	*Non-Op*	93.3	116.5		32.6	
	*Difference*	38.1	28.9		12.4	
	*p-value*	<0.01	<0.01		0.03	

RSA: reverse shoulder arthroplasty.

a The Constant Murley (CM) scale was used to quantitatively analyse internal rotation: lateral thigh = 0, buttock = 2, lumbosacral junction = 4, waist = 6, 12th dorsal vertebra = 8 and interscapular region = 10 points.

**Fig. 6:**
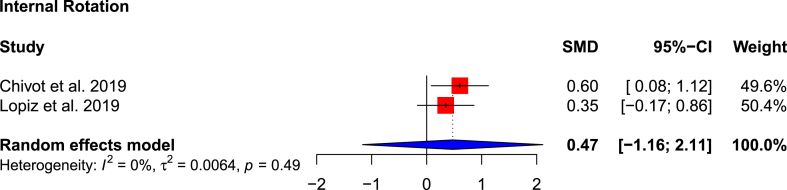
Forest plot showing pooled internal rotation movement.

Internal rotation was reported by two studies,^1^^,^^22^ with an average score of 5.44 in the RSA cohort according to the Constant-Murley scale,^14^ and 4.28 in the non-operative cohort. The difference is not statistically significant (SMD: 0.47; 95 % CI: −1.16-2.11; p = 0.169; I^2^ = 0 %) (Fig. 6).

e. Complications

**Fig. 7:**
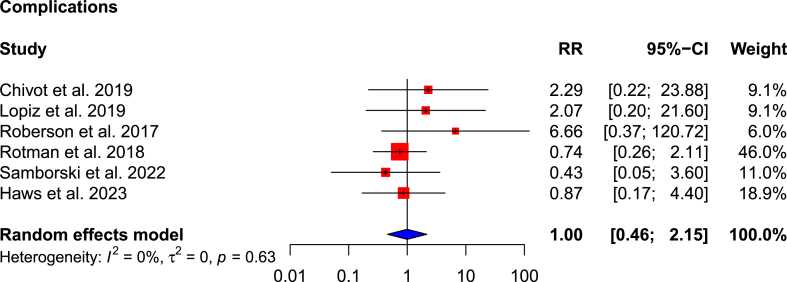
Forest plot of overall complications.

**Fig. 8:**
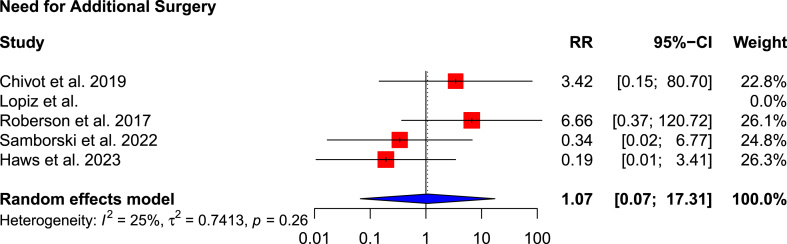
Forest plot of need for additional surgery.

The risk ratio of complications and the need for subsequent surgery in the RSA cohort compared to non-operative cohort is 1.00 (95 % CI: 0.46–2.15; p = 0.993; I^2^ = 0 %) (Fig. 7) and 1.07 (95 % CI: 0.07–17.31; p = 0.640; I^2^ = 25 %) (Fig. 8), respectively. This means that there is no statistically significant difference in the overall complication rate (8.2 % versus 6.0 %), but the RSA cohort has a higher risk of needing subsequent surgery (3.7 % versus 2.8 %) (Table 4).

**Table 4:** Complications.

Author		Overall (%)	Subsequent Surgery (%)	Infection (%)	Dislocation (%)	Adhesions (%)	Nerve Injury (%)	Mortality (%)	Nonunion (%)
Chivot et al., 2019	*RSA*	7	3	0	7	0	0	N/A	0
	*Non-Op*	3	0		0				3
	*Difference*	4	7		7				3
	*p-value*	N/A	N/A		N/A				N/A
Lopiz et al., 2019	*RSA*	6.9	0	0	0	0	6.9	N/A	0
	*Non-Op*	3					0		3
	*Difference*	3.9					6.9		3
	*p-value*	N/A					N/A		N/A
Rotman et al., 2018	*RSA*	8.1	N/A	0	0	0	0	8.1	N/A
	*Non-Op*	10.8						10.8	
	*Difference*	2.7						2.7	
	*p-value*	0.56						0.56	
Roberson et al., 2017	*RSA*	15	15	3.3	3.3	3.3	0	N/A	0
	*Non-Op*	0	0	0	0	0			
	*Difference*	15	15	3.3	3.3	3.3			
	*p-value*	0.08	0.08	N/A	N/A	N/A			
Samborski et al., 2022	*RSA*	4.2	0	0	0	0	0	N/A	0
	*Non-Op*	7.4	4.9						2.5
	*Difference*	3.2	4.9						2.5
	*p-value*	<0.01	<0.01						N/A
Haws et al., 2023	*RSA*	7.7	0	0	0	3.8	3.8	N/A	0
	*Non-Op*	8.9	8.9			0	0		7
	*Difference*	1.2	8.9			3.8	3.8		7
	*p-value*	0.36	N/A			N/A	N/A		N/A

Roberson et al.^23^ reported no complications in the non-operative cohort but three complications (infection, adhesions, dislocation) in the RSA cohort. They all required subsequent surgery, and was treated by debridement/irrigation, arthroscopic lysis, and open reduction, respectively. Chivot et al.^22^ reported two traumatic dislocations (one after cardiac syncope and another after urosepsis). Both required reoperation with removal of the implant. Lopiz et al.^1^ reported two cases of suprascapular nerve injury in the RSA cohort, and one case of non-union in the non-operative cohort. The only outcome measure reported by Rotman et al.^24^ was mortality at one year follow-up, and this was the only study to report this outcome measure. The mortality rate was greater in the non-operative cohort than the RSA cohort (10.8 % vs 8.1 %).

f. Bias

Risk of bias in the five non-randomised studies was assessed by ROBINS-I (Supplementary Fig. 1). Overall, there was moderate risk of bias in three^13^^,^^23^^,^^25^ out of the five studies. All studies apart from one^24^ had some bias in measurement of outcome since PROMs are subjective in nature, and some studies did not clearly define the outcome measures they were assessing. One study had bias in selection of participants since the exclusion criteria was not mentioned.^23^ Three studies had some bias due to deviation from intervention, since the surgical and rehabilitation technique was not described.^13^^,^^24^^,^^25^

Risk of bias in the RCT conducted by Lopiz et al.^1^ was assessed using ROB 2.0 (Supplementary Fig. 2). There was overall moderate risk of bias. There was not enough description of the randomisation process, such as how allocation sequences are generated, who is performing the randomisation process, and whether they are part of the trial analysis team. Treatments were not able to be concealed hence blinding of clinicians or participants was not possible, leading to potential bias in measurement of outcome. There was no mention of ways to increase the retention rate of participants, however the dropout rate was only 4.8 % (3/62).

Publication bias was assessed using the funnel plot (Supplementary Fig. 3). As shown by Egger's test, there was no statistically significant publication bias (p = 0.37, intercept: −1.051; 95 % CI: −3.1 to 1).

## 4 Discussion

The results of this review demonstrate that in the short-term (26.0 months follow-up), elderly patients with complex PHFs being treated with RSA may have decreased pain, increased Constant-Murley scores, and increased ROM compared with patients treated non-operatively. There is no significant difference in the rate of complications between the two cohorts.

### 4.1 Functional outcomes

Although most reported PROMs were numerically better in the RSA cohort than the non-operative cohort, none of the pooled results were statistically significant. Several of the individual results from included studies were statistically significant, e.g. the improvement in Constant-Murley score reported in Chivot et al. (76.8–82.1; p = 0.03).^22^ As reported in a multicentre study by Simovitch et al., the minimally clinically important difference (MCID) for the Constant-Murley score is 5.7 ± 1.9.^26^ Hence, despite being statistically insignificant (p = 0.071), the improvement in Constant-Murley score of 6.0 at twelve months follow-up reported in Lopiz et al. could be clinically important for a few individuals. Nevertheless, the sample size of the study is small, and no power analysis was performed in the study. Furthermore, a more recent study suggested a MCID of 9.3 at twelve months follow-up.^27^

VAS pain scores were noted to be significantly better in the RSA cohort in three studies.^1^^,^^13^^,^^25^ Unlike other studies comparing hemiarthroplasty with non-operative treatment,^28^ which found a difference in scores at 3 months follow-up but not at 12 months, no difference was found at 3 months follow-up, but a statistically significant difference was found at 12 months.^1^^,^^13^^,^^25^ Furthermore, the difference in VAS scores is only absent at 3 months follow-up, but present at all other stages of follow-up. The difference in VAS scores is most significant early in recovery, but gradually converged at 1 year follow-up, although still remaining statistically significant.^13^^,^^25^ This may form an important part of discussions with patients, regarding to reduced usage of painkillers and earlier return to sport or activities of daily living (ADLs). PROMIS pain scores were reported in two studies, one of which found significantly better scores in the RSA cohort than non-operative cohort at each stage of follow-up.^13^ Nevertheless, all the differences in VAS scores were <0.7, which is below the MCID of 1.6 ± 0.3,^26^ and may not be clinically relevant. Roberson et al.^23^ reported better pain scores in the non-operative cohort, and equal ASES scores between the two cohorts (statistically not significant). However, there might be selection bias in the study since the non-operative treatment group consisted of patients were who were initially offered a reverse prosthesis, but then declined.

Complex PHFs managed with RSA showed improved ROM compared to non-operative management at final follow-up. This was seen in both active movements (forward flexion, external rotation, abduction) as well as passive forward flexion. PROMIS physical function scores were reported in two studies, both of which showed significant better results in the RSA cohort than the non-operative cohort from three months follow-up onwards.^13^^,^^25^ This could be because most rehabilitation protocols do not include active ROM early in recovery, hence there will be little difference in active ROM between cohorts.

### 4.2 Complications

This review found no difference in the risk of complications between the RSA cohort and the non-operative cohort. This agrees with a recently published meta-analysis comparing arthroplasty (including hemiarthroplasty) and non-operative treatment.^12^ Malunion, defined as a head-shaft angle (HSA) <120° or >150° or head-shaft translation >5 mm^1^, was not included in the analysis of complications. Lopiz et al.^1^ reported that all patients in their non-operative cohort had malunion whilst none were found in the RSA cohort. Samborski reported a 77.5 % malunion rate in the non-operative cohort. Risk of needing subsequent surgery was also greater in the RSA cohort, but was statistically insignificant. This may not be surprising in clinical practice since more complex fracture are harder to manage surgically, and are more likely to need additional procedures to manage complications.^7^ However, more studies are needed to understand if the risk of needing subsequent surgery is influenced by clinical decision making that differs between surgeons.

The validity of results from this review is limited by the small sample size and differing definitions of complications. Samborski et al.^25^ was the only included study that gave definitions of non-union or malunion. Furthermore, the follow-up time in most included studies was short, and complications such as scapular notching or implant loosening were not able to be assessed. Further studies are needed to determine the mid- and long-term survivorship of RSA protheses for complex PHFs in the elderly.

Rotman et al.^24^ was the only study to report mortality rate, which was lower in the RSA cohort than the non-operative cohort at both one month (p = 0.61) and one year follow-up (p = 0.56). The mortality rate increased from one month to one year in both cohorts. Studies have reported mortality rate to continue increasing after the one year mark,^29^ whereas in spine and hip fractures, mortality declines after the 1st year.^30^ As suggested by Johnell et al., the main cause of mortality in hip fractures is morbidity related to the fracture, and a decline in mortality corresponds to a decrease in deaths causally related to the fracture.^30^^,^^31^ In PHFs, the increasing mortality rate after fracture resolution may be a reflection of frailty in this population and other factors not associated with fracture morbidity.^24^ Surgery might be able to improve an elderly patient's life beyond the clinical and functional parameters assessed in this review. Given the prevalence of osteoporosis, osteoarthritis, and sarcopenia in this population, non-operative treatment could exacerbate these conditions, leading to further fragility fractures and increased morbidity in future.

### 4.3 Directions for future studies

A major debate amongst upper limb orthopaedic surgeons is whether or not to treat proximal humerus fractures, especially in the elderly. Unlike displaced femoral or tibial fractures, there is no clear cut decision-making algorithm regarding the treatment of osteoporotic PHFs. This may be because of low-quality evidence in this area due to problems with study design, such as heterogenous cohorts in terms of age and fracture type, presence of interobserver differences in the assessment of PROMs, or selection bias. Nevertheless, level I studies have been published, with the latest being the PROFHER trial,^4^ a multi-centre RCT performed in the UK. Despite the conclusion that there are no differences in terms of functional and clinical outcomes between surgical and non-operative treatment, there are numerous flaws that bias the results. A wide range of ages (16–66) were included, and only 4.4 % (11/250) were Neer four-part PHFs, limiting external validity. Over the two year follow-up period, 125 patients were operated on by 66 surgeons, which is less than one case per surgeon each year. This is likely a huge underrepresentation of the PHFs occurring at the participating centres during the timeframe. Furthermore, some patients were excluded for questionable reasons, e.g. patients not being randomised because they had ‘clear indications for surgery’ (n = 87), ‘associated dislocations’ (n = 100). A Cochrane review^32^ concluded that surgery does not result in better outcomes than non-surgical treatment. However, as pointed out by Sabharwal et al.,^7^ differences in fracture type and surgical techniques leads to outcomes that differ from those obtained from generalised analyses. It is hence inappropriate to enquire whether ‘to operate or not operate’, but rather ‘on whom to operate’,^33^ for which the Cochrane review concludes that not enough high-quality evidence exists to answer this.^32^

There are ongoing trials, with protocols published, comparing RSA versus non‐surgical treatment.^6^^,^^34^^,^^35^ Notably, the PROFHER-2 trial^6^ seeks to remedy the defects of the previous PROFHER trial, and will only include adults over 65 with Neer 3- or 4-part fractures.

In this review of six included studies, five utilised the Neer classification, whilst Samborski et al.^25^ utilised the AO/OTA system. Standardisation of fracture classification will help provide clinically applicable findings to inform decision making. Amongst the seven ongoing RCTs registered in the World Health Organization's International Clinical Trials Registry Portal, the Neer (NCT02362100, NCT01246167, NCT00999193, NCT00835562, NCT0818987), AO/OTA (NCT00863473) and Hertel (NTR2040) classification systems are all being used. Given the fact that inter- and intraobserver reliability differs between classification systems, it is important to have a homogenous classification system in trials to make unbiased comparisons.^36^

Although this review includes studies that have defined the cohorts to be ‘elderly’, the definition of ‘elderly’ differs. Although those aged 65 or over is a common definition,^13^^,^^23^ some use the cut off age of 60,^25^ 70,^22^ 75,^24^ or 80.^1^ Furthermore, the concept of elderly is not simply about age but also encompasses the concepts of frailty and associated comorbidities. The CCI index allows us to not only quantify comorbidities in the elderly but also estimate the 10-year mortality rate. This was only reported in all included studies apart from one.^22^ Frailty also gives us an idea of physiological reserve, and the ability to cope with procedures such as RSA.

### 4.4 Limitations

This review showed low to moderate between-study heterogeneity in effect sizes, although Cochran's Q test was not statistically significant for any pooled effect size. This suggests a low to moderate likelihood that there are differences between studies that are not due to chance, influencing the conclusions generated from this review. This may be due to varying surgical techniques and post-operative rehabilitation protocols. There is heterogeneity in reported outcome measures and the lack of consideration of important PROMs to use. There is the need to develop a core outcome set, which is an ‘agreed standardised set of outcomes that should be reported, as a minimum in all clinical trials’.^37^ This would ensure that every trial is able to contribute useful data to the development of robust evidence based clinical guidelines. The ‘core outcome set for proximal humerus fractures (COSH)’^38^ trial was in progress but unfortunately was terminated. Equally as important is to develop a core set of clinically unfavourable events, which was done using an international Delphi consensus process by Audige et al.^39^

The follow-up period in four of the six included studies is twelve months, which precluded the analysis of long-term complications such as osteonecrosis, post-traumatic arthritis, scapular notching, or implant loosening. Nevertheless, this review investigated the use of arthroplasty for a fracture in elderly patients with comorbidities, hence short-term results may prove adequate to determine their short term quality of life. In general, the sample size was small, increasing the chance of a type II error. Only one study was randomised in design, hence there is intrinsic selection bias in most of the included studies and a large number of patients lost to follow-up. Treatment plans were made subjectively based on an individual surgeon's decision. This decreases the statistical power of conclusions drawn from the data. Key terms such as malunion and non-union were not defined in most studies, and some terms were used interchangeably, e.g. reoperation and revision surgery. Most of the included studies were performed in a single centre. Multicentre trials such as PROFHER will increase the external validity of conclusions. This is important in surgical trials, since surgical experience and expertise varies, and performance bias may hamper the validity of results.

## 5 Conclusion

This review demonstrates that elderly patients with complex PHFs being treated with RSA may have decreased pain, increased Constant-Murley scores, and increased ROM in the short-term compared with patients treated non-operatively. There is no difference in overall complication rate between the two cohorts. The observed clinical difference was small, and a few variables did not reach the MCID. Given the results of previous systematic reviews showing that acute RSA leads to improved clinical and functional outcomes compared to delayed RSA,^2^ RSA should be considered for those with increased functional demands. There was low to moderate between-study heterogeneity in effect sizes and the retrospective nature of included studies may limit the clinical applications of conclusions obtained in this review. Nevertheless, this review creates a baseline from which future prospective trials can be built on to create guidelines to aid clinical decision making for the optimal treatment for complex PHFs in the elderly. There are currently four clinical trials in progress comparing RSA with non-operative treatment in the elderly; the results are keenly awaited.

## CRediT authorship contribution statement

**Victor Yan Zhe Lu:** Data curation, Formal analysis, writing, review of manuscript. **Halia Shah:** Data curation, writing, review of manuscript. **Zainab Alshaber:** Data curation, writing, review of manuscript. **Aaron Limonard:** Writing, review of manuscript. **Peter Domos:** Conceptualization, review of manuscript.

## Patient's consent

This manuscript is a review article and does not require patient consent.

## Availability of data and material

The authors confirm that the data supporting the findings of this study are available within the article [and/or] its supplementary materials.

## Consent to publication

All authors have reviewed the final version and have consented to publication.

## Ethics approval

This is a systematic review, and no ethical approval is required.

## Ethics statement

This is a systematic review, and no ethical approval is required.

## Funding statement

The authors did not receive support from any organization for the submitted work.

## Declaration of competing interest

The authors declare that they have no known competing financial interests or personal relationships that could have appeared to influence the work reported in this paper.
