# Supplementary figures and images for: Non-operative versus reverse shoulder arthroplasty for the treatment of 3- or 4-part proximal humeral fractures: A systematic review and meta-analysis
Source: J Clin Orthop Trauma. 2025 Mar 22;65:102982. doi: 10.1016/j.jcot.2025.102982 (PMC11986627; doi:10.1016/j.jcot.2025.102982)

## Summary of ROBINS-I assessments

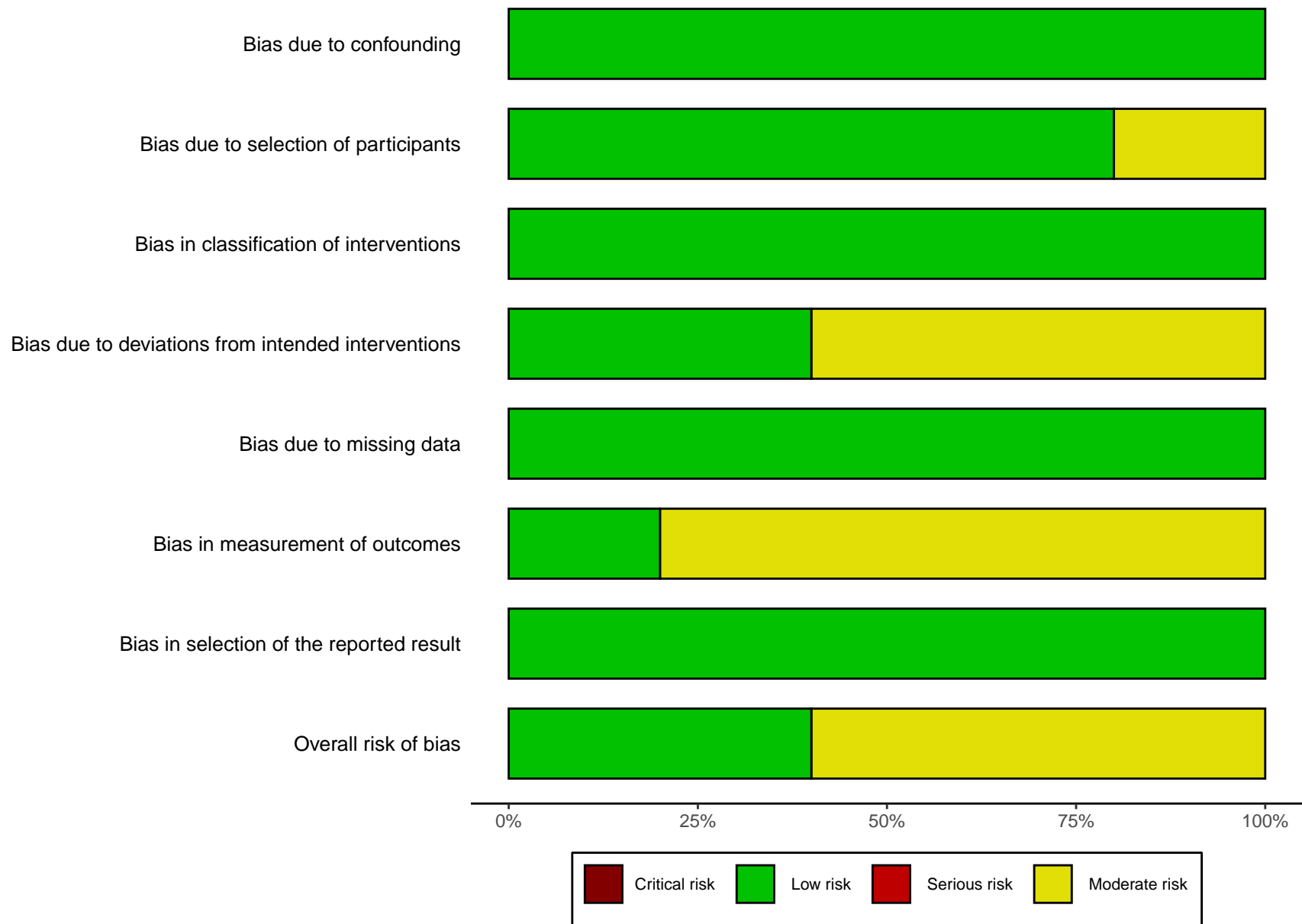

Supplement: Multimedia component 1 [file mmc1.pdf]

## Summary of ROB 2.0 assessments

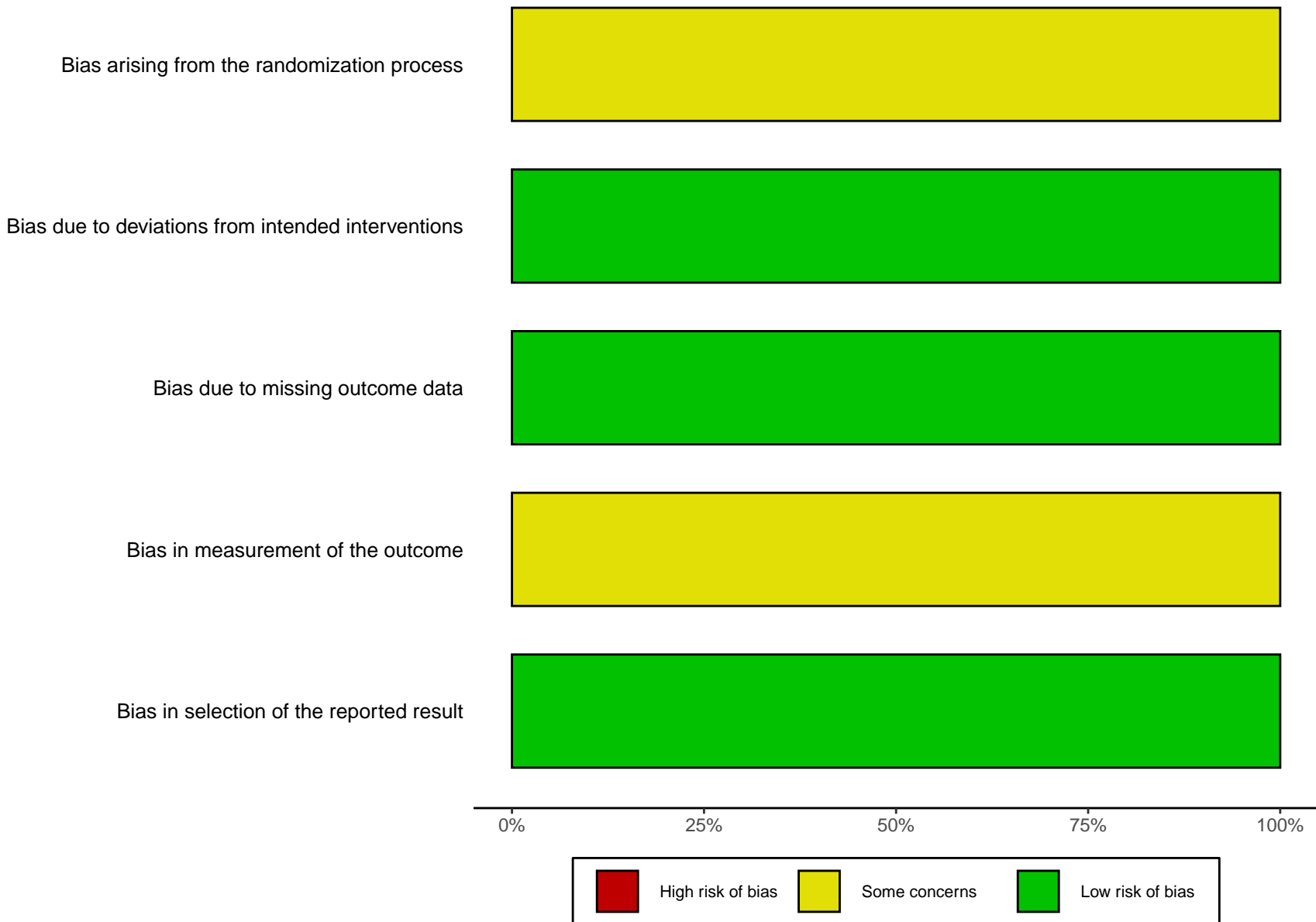

Supplement: Multimedia component 2 [file mmc2.pdf]

# Contour-Enhanced Funnel Plot for Publication Bias

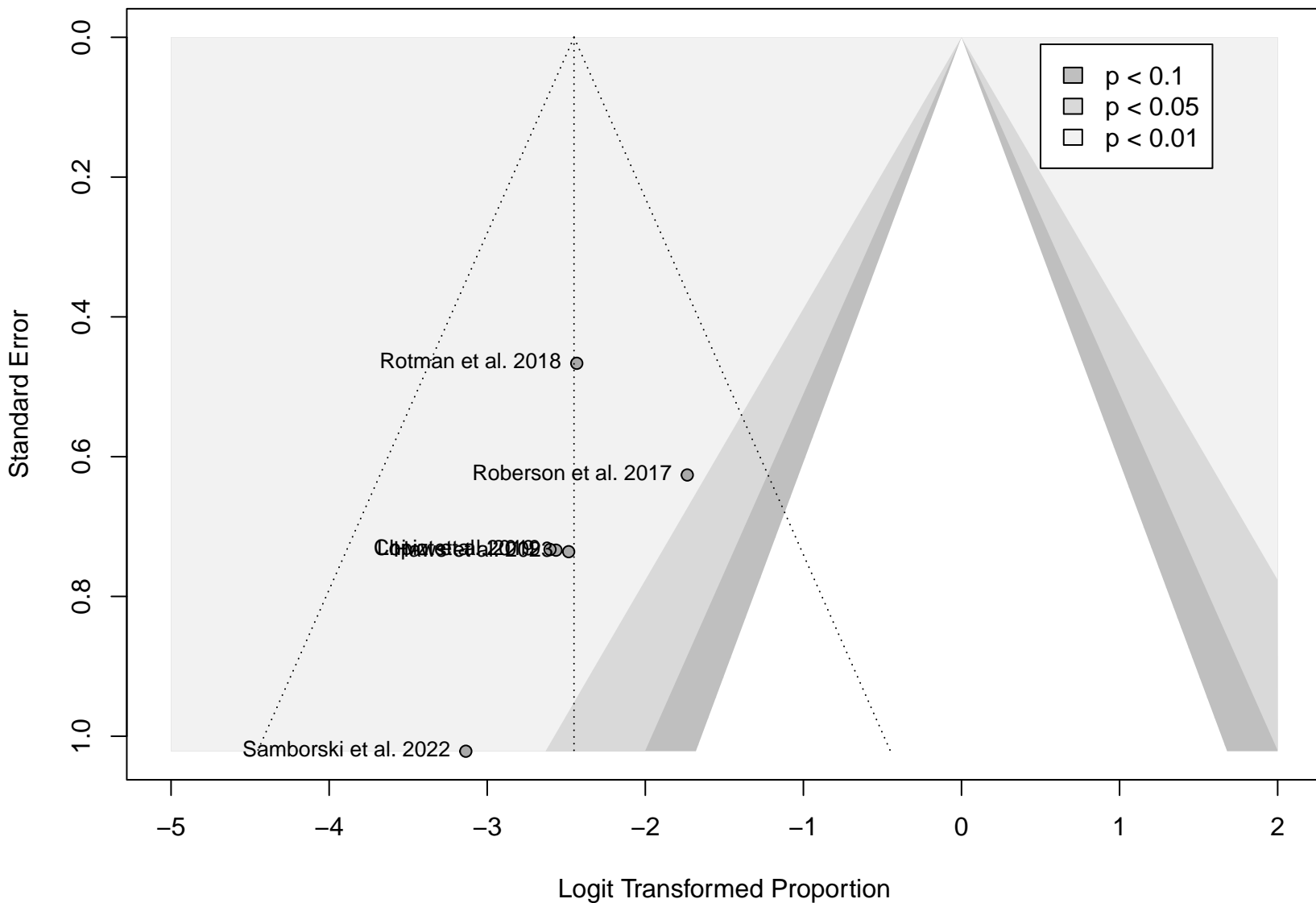

Supplement: Multimedia component 3 [file mmc3.pdf]
