# Supplementary material for: Non-operative versus reverse shoulder arthroplasty for the treatment of 3- or 4-part proximal humeral fractures: A systematic review and meta-analysis
Source: J Clin Orthop Trauma. 2025 Mar 22;65:102982. doi: 10.1016/j.jcot.2025.102982 (PMC11986627; doi:10.1016/j.jcot.2025.102982)
Supplement: Multimedia component 4 [file mmc4.docx]

| **Supplementary Table 1**: Detailed search strategy | | | |
| --- | --- | --- | --- |
| **Proximal Humerus Fracture:** | **Reverse Shoulder Arthroplasty:** | **RSA:** |  |
| 1) shoulder fracture | 6) reverse total shoulder arthroplasty | 11) Acute | 17) 5 AND 10 AND 16 |
| 2) humerus fracture | 7) reverse total shoulder replacement | 12) Delayed |  |
| 3) proximal humerus fracture | 8) reverse shoulder arthroplasty | 13) Revision |  |
| 4) proximal humerus | 9) reverse shoulder replacement | 14) Primary |  |
| 5) 1 OR 2 OR 3 OR 4 | 10) 6 OR 7 OR 8 OR 9 | 15) Secondary |  |
|  |  | 16) 11 OR 12 OR 13 OR 14 OR 15 |  |
| Databases searched: Ovid EMBASE, PubMed MEDLINE, Web of Science, and Scopus.  Databases were searched from inception to October 10^th^ 2023. No filters or limits were used. | | | |
