# Supplementary material for: Non-operative versus reverse shoulder arthroplasty for the treatment of 3- or 4-part proximal humeral fractures: A systematic review and meta-analysis
Source: J Clin Orthop Trauma. 2025 Mar 22;65:102982. doi: 10.1016/j.jcot.2025.102982 (PMC11986627; doi:10.1016/j.jcot.2025.102982)
Supplement: Multimedia component 5 [file mmc5.docx]

| **Supplementary Table 2**: PICOS inclusion and exclusion criteria for study selection. | | |
| --- | --- | --- |
| **Domain** | **Inclusion Criteria** | **Exclusion Criteria** |
| **Population** | Clinical studies on human populations with a proximal humerus fracture.  Mean age ≥65 | Patients with preoperative glenohumeral joint infection  Patients with pathological fracture  Patients with concomitant neurological lesion of the fractured arm  Patients with ipsilateral upper limb fracture  Patients with glenohumeral joint arthritis  Average age of patient cohort is below 65 years old. |
| **Intervention** | Studies utilising reverse shoulder arthroplasty (RSA) or non-operative management to treat 3- or 4-part proximal humerus fractures. | Studies utilising RSA or non-operative management for indications other than trauma. |
| **Comparison** | Studies comparing RSA with non-operative management for the treatment of 3- or 4-part proximal humeral fractures. | Studies that include only RSA as a treatment option or only non-operative management.  Studies comparing patient cohorts using RSA with other surgical procedures (e.g. ORIF or HA). |
| **Outcome** | Studies reporting clinical and functional outcomes, including PROMs, need for reoperation, radiological findings, post-operative complications, and mortality. | Studies where no outcome measures are directly related to efficacy of RSA or non-operative management for the treatment of proximal humerus fractures. |
| **Study Type** | Primary studies written in English with full-text available.  Minimum follow-up time of 12 months. | Case reports, abstracts, reviews.  Studies not written in English.  Papers describing surgical technique only.  *In vitro* studies  *Ex vivo* studies  Animal studies |
